# The introduction of an N-glycosylation site into prochymosin greatly enhances its production and secretion by *Pichia pastoris*

**DOI:** 10.1186/s12934-022-01904-3

**Published:** 2022-08-30

**Authors:** Nan Wang, Caifeng Yang, Huakang Peng, Wenfang Guo, Mengqi Wang, Gangqiang Li, Dehu Liu

**Affiliations:** Biotechnology Research Institute, Chinese Academy of Agricultural Sciences, Beijing, 100081 China

**Keywords:** N-glycosylation, Prochymosin, Secretion, *Pichia pastoris*, Differential interacting proteins

## Abstract

**Background:**

N-glycosylation is one of the most important post-translational modifications. Many studies have shown that N-glycosylation has a significant effect on the secretion level of heterologous glycoproteins in yeast cells. However, there have been few studies reporting a clear and unified explanation for the intracellular mechanism that N-glycosylation affect the secretion of heterologous glycoproteins so far. *Pichia pastoris* is an important microbial cell factory producing heterologous protein. It is of great significance to study the effect of N-glycosylation on the secretion level of heterologous protein. Camel chymosin is a glycoprotein with higher application potential in cheese manufacturing industry. We have expressed camel prochymosin in *P. pastoris* GS115, but the lower secretion level limits its industrial application. This study attempts to increase the secretion level of prochymosin through N-glycosylation, and explore the molecular mechanism of N-glycosylation affecting secretion.

**Results:**

Adding an N-glycosylation site at the 34th amino acid of the propeptide of prochymosin significantly increased its secretion in *P. pastoris*. N-glycosylation improved the thermostability of prochymosin without affecting the enzymatic activity. Immunoprecipitation coupled to mass spectrometry (IP-MS) analysis showed that compared with the wild prochymosin (chy), the number of proteins interacting with N-glycosylated mutant (chy34) decreased, and all differential interacting proteins (DIPs) were down-regulated in chy34-GS115 cell. The DIPs in endoplasmic reticulum were mainly concentrated in the misfolded protein pathway. Among the five DIPs in this pathway, overexpression of BiP significantly increased the secretion of chy. The knockout of the possible misfolded protein recognition elements, UDP-glycose:glycoprotein glucosyltransferase 1 and 2 (UGGT1/2) had no effect on the growth of yeast cells and the secretion of prochymosin.

**Conclusions:**

In conclusion, N-glycosylation increased the secretion of prochymosin in *P. pastoris* trough the adjustment of intracellular interacted proteins. The results of our study may help to elucidate the molecular mechanism of N-glycosylation affecting secretion and provide a new research method to improve the secretion of heterologous glycoprotein in *P. pastoris*.

**Supplementary Information:**

The online version contains supplementary material available at 10.1186/s12934-022-01904-3.

## Background

In eukaryotic cells, N-glycosylation is one of the most important post-translational modifications and plays an important role in biological processes [1]. N-glycosylation affects biological activity, thermostability, protease tolerance, signal transduction, localization, and the secretion of glycoproteins [2–9]. N-glycosylation of eukaryotic cells is a conservative process. In the endoplasmic reticulum (ER), oligosaccharide transferase (OST), an ER membrane-localized complex, transfers an oligosaccharide (Glc3Man9GlcNAc2) to the Asn residue within the Asn-Xxx-Ser/Thr (where Xxx is any amino acid except Pro) recognition sequence in a nascent protein. Then, the outer three glucose molecules of the oligosaccharide are successively cleaved by α-glucosidase I and II. If the protein folding is completed quickly, it will be transported to the Golgi apparatus via vesicular trafficking for further glycosylation [10, 11], and finally, the glycoproteins are localized to a certain organelle or secreted to the outside of the cell. In contrast, if the glycoproteins does not fold properly, many studies inferred that the UDP-glucose: glucoprotein glucosyltransferase (UGGTs, including UGGT1 and UGGT2) will recognize it and adds a single glucose to the N-glycan [12, 13]. The GlcMan9GlcNAc2 acts as a signal to recruit the lectins calnexin (CNX) and other molecular chaperones, like Protein disulfide isomerase (PDI), to help the glycoproteins complete the folding, while preventing the non-folded glycoproteins from leaving the ER. For glycoproteins that cannot complete the correct folding over a long period of time, α-mannosidase will remove mannose and the remaining N-glycan as a signal to enter the degradation pathway. This process is call ER-associated degradation (ERAD) [14]. Meanwhile, the BiP, a molecular chaperone in the ER lumen, has been shown to interacts with unfolded proteins and participants in the ERAD [15, 16]. In the glycoprotein processing, it is worth noting that the function of UGGTs in the important microbial cell factory *Pichia pastoris* (*P. pastoris*) has not been confirmed.

Many studies have shown that N-glycosylation has a significant effect on the secretion level of heterologous glycoproteins in yeast cells [6, 7]. The position of glycosylation in glycoprotein plays a key role in this process [17]. Most N-glycosylation can improve secretion of glycoproteins [18–20], but there are also cases where the secretion is reduced [21] or not changed [22, 23]. However, there have been no studies reporting a clear analysis of and unified explanation for the intracellular mechanism of N-glycosylation affecting the secretion of heterologous glycoproteins. *P. pastoris* is an important microbial cell factory producing heterologous protein. It is of great significance to study the effect of N-glycosylation on the secretion level of heterologous glycoprotein.

Chymosin is an important enzyme in cheese manufacturing industry. Compared with widely used bovine chymosin, camel chymosin has higher clotting activity, specificity of protease activity, and thermostability [24, 25]. These characteristics make camel chymosin more advantageous in reducing the cost of production, transportation, storage and improving the flavor of cheese. The inactive camel prochymosin contains 365 amino acid residues. Its N-terminal has a propeptide of 42 residues, which is remove in an acidic environment, thereby leading to activation of the enzyme, this process known as autocatalytic cleavage. In our previous study, recombinant *P. pastoris* GS115 host expressing camel prochymosin was constructed [26], and the secretion level was approximately 37 mg/L under shaking flask conditions. This secreted level is too low to meet the needs of industrial application. The purpose of this study is to increase the secretion of prochymosin in *P. pastoris* as well as explore the intracellular molecular mechanism of N-glycosylation affecting secretion.

## Results

### N-glycosylation improved thermostability of prochymosin and increased secretion in *P. patoris*

**Fig. 1:**
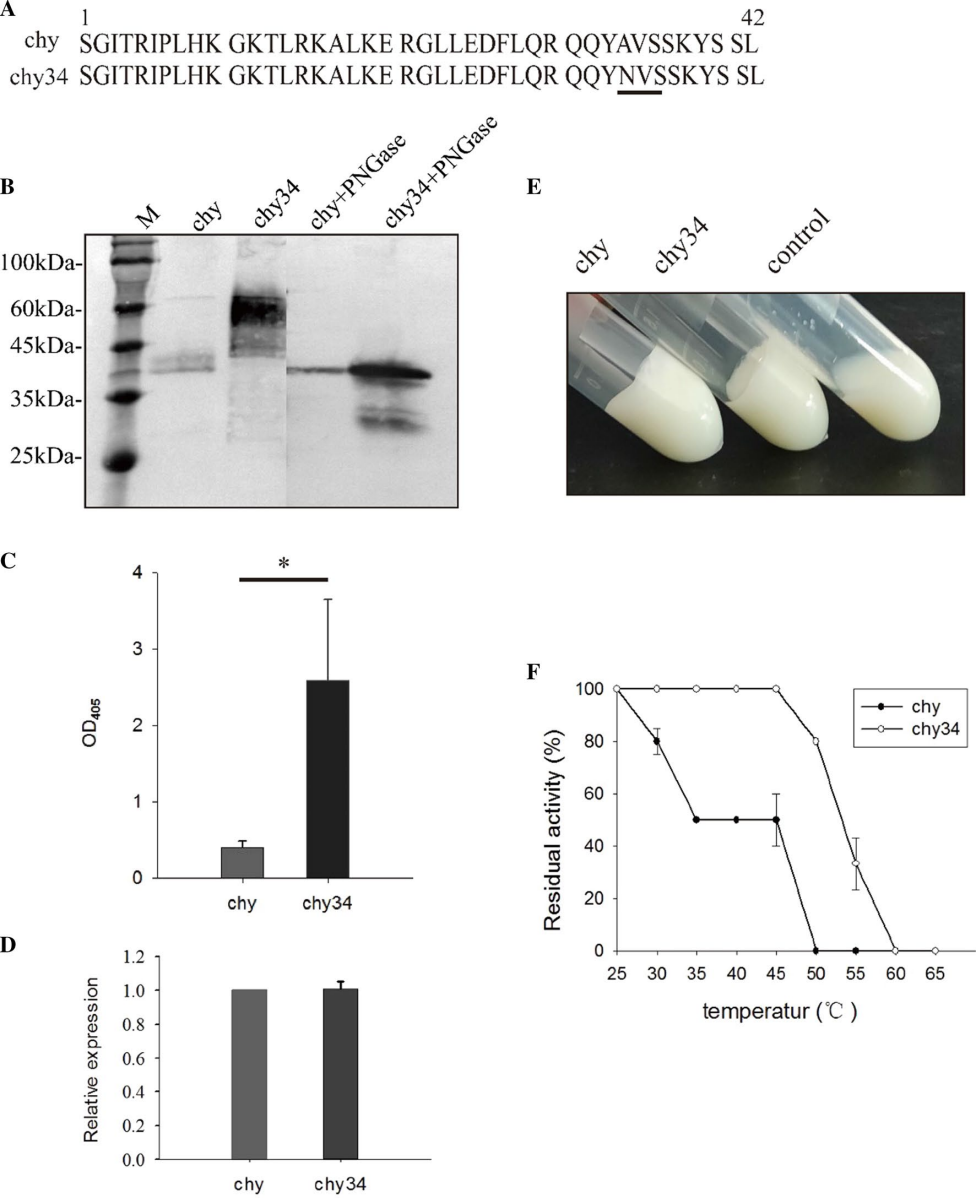
The effect of N-glycosylation on molecular weight, autocatalytic cleavage activity, thermal stability of prochymosin and the secretion in *P. pastoris* GS115. **A** Mutant site of N-glycosylation in the propeptide of prochymosin, conservated N-glycosylated sequences were indicated by underlines; **B** molecular weight and de-glycosylation analysis of chy and chy34 by Western-blot; **C** Determination of the secretion of chy and chy34 by ELISA; **D** The mRNA levels of chy and chy34 in host cells determined by qRT-PCR; **E** Analysis of autocatalytic cleavage of the propeptide by confirming the milk-clotting acidity, The control was the supernatant of GS115 culture carried the pPIC9K vector; **F** Thermostability of chy and chy34 when stored at different temperatures for 8 h prior to enzymatic activity assay. Error bars indicate the standard deviation of tree independent experiments, student’s t-test was used to compare difference between two groups, *P < 0.05

In order to increase the secretion of prochymosin in *P. pastoris* without affecting the clotting activity, an N-glycosylated conserved sequence at amino acid position 34–36 of the propeptide (Fig. 1A) was constructed by site-directed mutation (A34N). The mutant prochymosin (chy34) could be secrete in *P. pastoris*, and its molecular weight becomes larger (from ~ 40 kDa to ~ 60 kDa). After treatment with PNGase F, the molecular weight of chy34 decreased to the same as that of wild prochymosin (chy), indicating that the glycosylation site was N-glycosylated (Fig. 1B). The secretion level of chy34 was significantly higher than that of chy (Fig. 1C). There was no significant difference in mRNA level (Fig. 1D), indicating that the difference in protein secretion was not caused by copy number. The enzymatic activity analysis showed that the N-glycosylation mutations in the propeptide region did not affect its autocatalytic cleavage activity (Fig. 1E). Chy and chy34 were incubated at different temperatures. After 8 h of incubation at 45 °C, the residual activity of chy decreased to about 50%, while chy34 remained fully active (Fig. 1F), indicating that N-glycosylation improved the thermostability of the prochymosin. This improvement of thermostability was further verified on the secondary and tertiary structure of protein by circular dichroism spectroscopy (CD) and fluorescence spectroscopy, respectively. The CD spectra (Additional file 1: Fig. S5A) revealed that there was no significant difference in the proportion of secondary structure between the chy and chy34 proteins. Fluorescence emission spectra (Additional file 1: Fig. S5B) showed that the maximum emission of chy34 had a slight blue shift, and the fluorescence density decreased, it indicated that the thermostability of chy34 was improved, which may be attributed to the effect of N-glycosylation on the tertiary structure of protein.

### Identification of differential proteins interacting with chy and chy34

In order to explore the molecular mechanism of N-glycosylation affecting the secretion of prochymonsin, the differential interacting proteins (DIPs) with chy and chy34 in two host cells were determined by immunoprecipitation coupled to mass spectrometry (IP-MS) analysis. First, the agarose beads combined with anti-prochymosin antibody were used to immunize and precipitate the proteins that directly or indirectly interacting with chy and chy34. Then two groups of proteins were analyzed by mass spectrometry. The results of mass spectrometry searched against the UniProt database. The DIPs were identified by comparing all the proteins obtained from the database. Then GO annotation, KEGG enrichment analysis and COG analysis were performed on the DIPs.

**Fig. 2:**
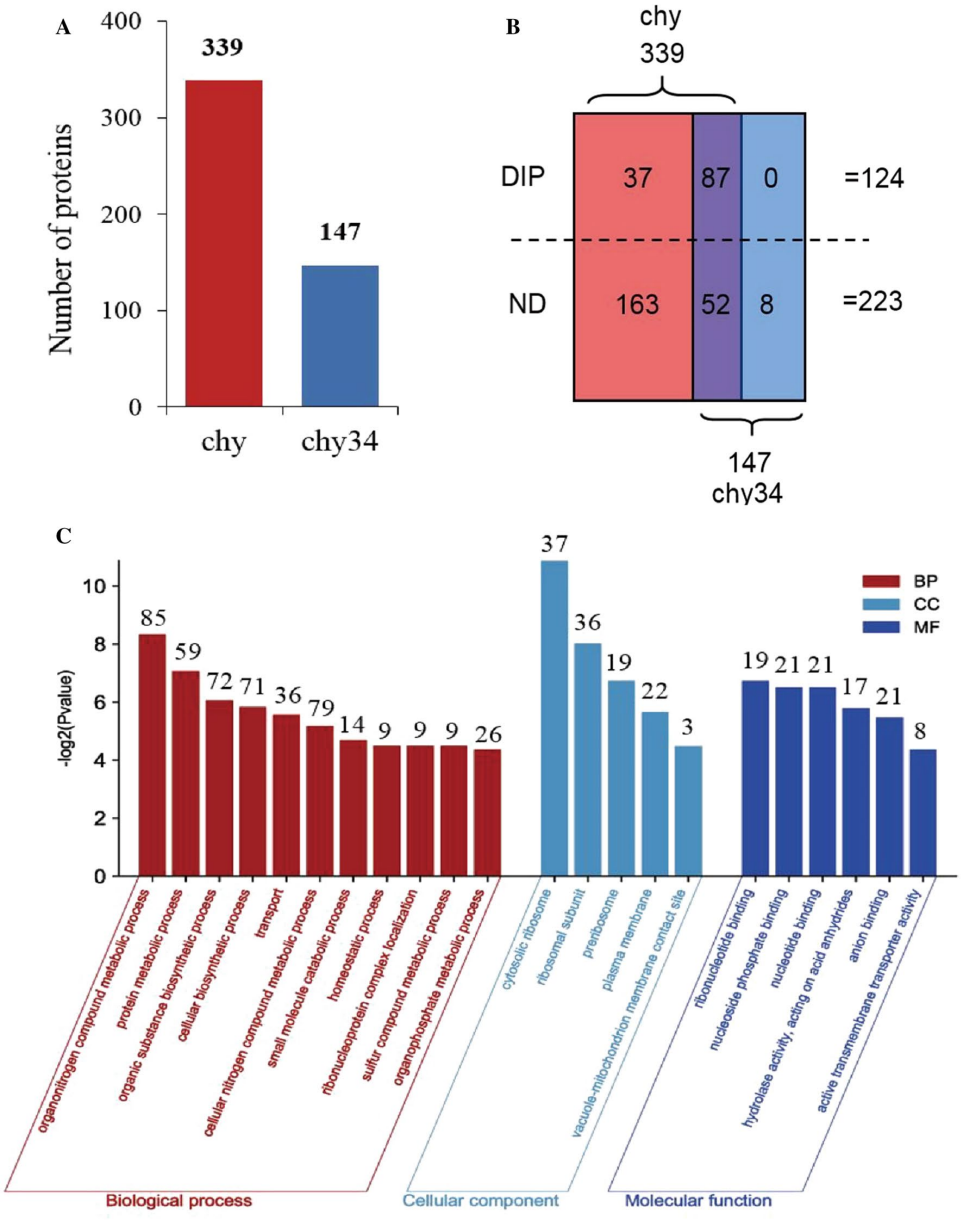

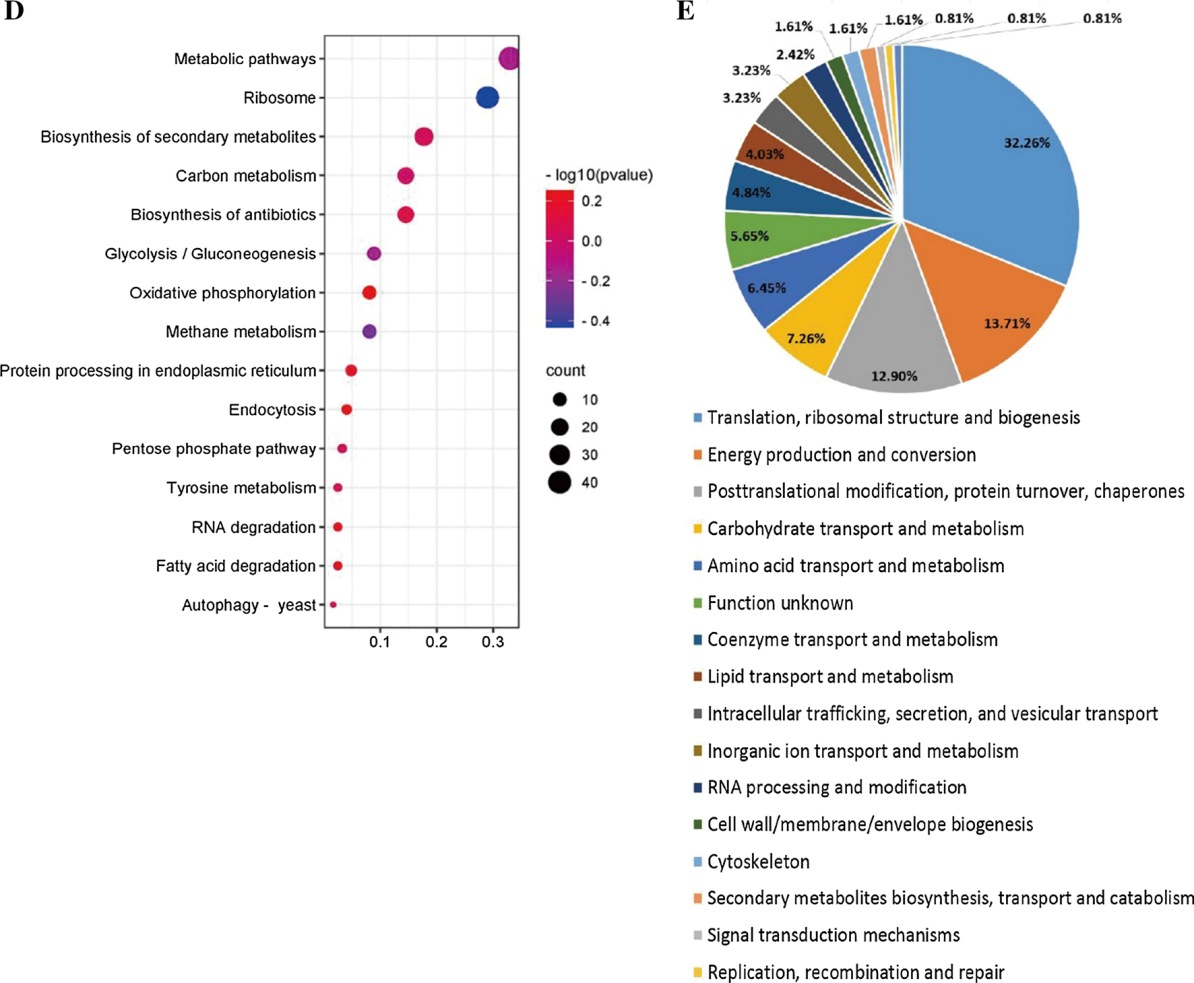
Bioinformatics analysis of IP-MS data. **A** Number of differential interacting proteins (DIPs); **B** Distribution of DIPs and no differentially interacting proteins (ND) in chy and chy34 samples; **C** GO function classification of DIPs; **D** KEGG enrichment analysis of DIPs; **E** COG analysis of DIPs. The terms of p Value < 0.05 were displayed

After the proteins sample obtained by immunoprecipitation were digested by trypsin, the peptide length is mainly between 8 and 20 (Fig. S1A), which conforms to the regular of trypsin digestion and meets the standard for mass spectrometry. By the detection of mass spectrometry, 147 proteins interacting with chy34 and 339 proteins interacting with chy were identified (Fig. 2A), and the number of spectra, peptides and proteins interacting with chy was higher than that of chy34 (Additional file 1: Fig. S1B). Taking the fold change ≥ 1.5 as the screening standard, 124 DIPs were obtained (Additional file 2: Table S1). All DIPs were down-regulated in chy34 (Additional file 1: Fig. S1C), which showed higher secretion in *P. pastoris*. Among the DIPs, 87 interacted with both chy and chy34, and 37 interacted only with chy (Fig. 2B). These results showed that the type and number of proteins interacting with chy were significantly more than those of chy34.

### Annotation and enrichment analyses of DIPs

GO annotation and enrichment analysis was performed on 124 DIPs. The top terms (P Value < 0.05) were ranked based on the P value. The top Biologycal Process (BP) terms were organonitrogen compound metabolic process, protein metabolic process, organic substance biosynthetic process, etc.; the top Cellular Component (CC) terms were cytosolic ribosome, ribosomal subunit, preribosome, etc.; the top Molecular Function (MF) terms were ribonucleotide binding, nucleoside phosphate binding, nucleotide binding etc. (Fig. 2C). The KEGG pathway enrichment analysis (Fig. 2D) showed that the DIPs were mostly involved in Ribosome, Glycolysis/Gluconeogenesis, Autophagy—yeast, Protein processing in endoplasmic reticulum, etc. COG analysis (Fig. 2E) showed that the functions of DIPs were enriched in Translation, ribosomal structure and biogenesis, Energy production and conversion, Posttranslational modification, protein turnover, chaperones, etc. From the annotation and enrichment analysis results of DIPs, it can be seen that these DIPs are mainly related to protein synthesis, metabolism, energy production/transformation and homeostasis maintenance.

**Fig. 3:**
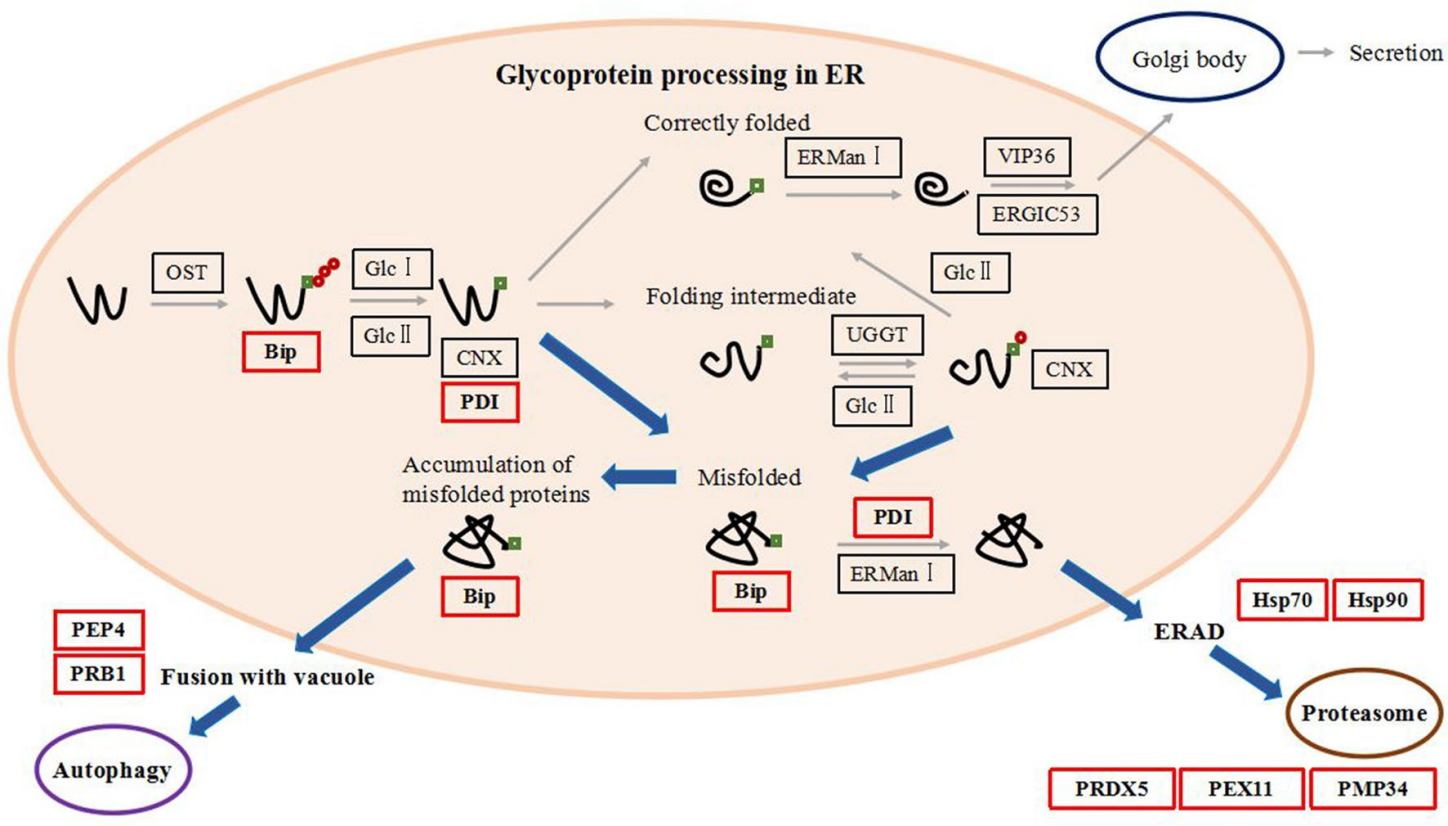
Diagram of glycoprotein processing in ER. The box represents the enzyme in the pathway, red means up-regulated enzyme in chy symple, black indicates ND or undetected proteins, green square represents mannose and red circle represents glucose. The blue arrows indicated ER-associated misfolded protein and degradation pathways

The translation, folding and modification of N-glycosylated proteins begin in the endoplasmic reticulum (ER). In the enriched KEGG pathways, there are six proteins in the ER related “protein processing in endoplasmic reticulum” pathway: PDI (Gene symbol: PAS_chr1-1_0160), Hsp90 (Gene symbol: PAS_chr1-4_0130), BiP (Gene symbol: PAS_chr2-1_0140), Hsp70 (Gene symbol: PAS_chr3_0230), Hsp70 (Gene symbol: PAS_chr3_0731), Hsp70 (Gene symbol: PAS_chr4_0552). These proteins are mainly involved in the metabolic pathway when glycoproteins misfolded (Fig. 3). The red rectangles in Fig. 3 mean up-regulated molecular chaperone or enzyme in chy sample. This indicated that in the ER, more molecular chaperones and enzymes were involved in the folding, secretion or degradation of wild chy. It also implied that after N-glycosylation of prochymosin, the proteins interacting with prochymosin in ER were down-regulated and the glycoprotein was released into the secretory pathway, that is, the added N-glycosylation changed the metabolic pathway of prochymosin in ER.

### BiP overexpression increased the secretion of chy

**Fig. 4:**
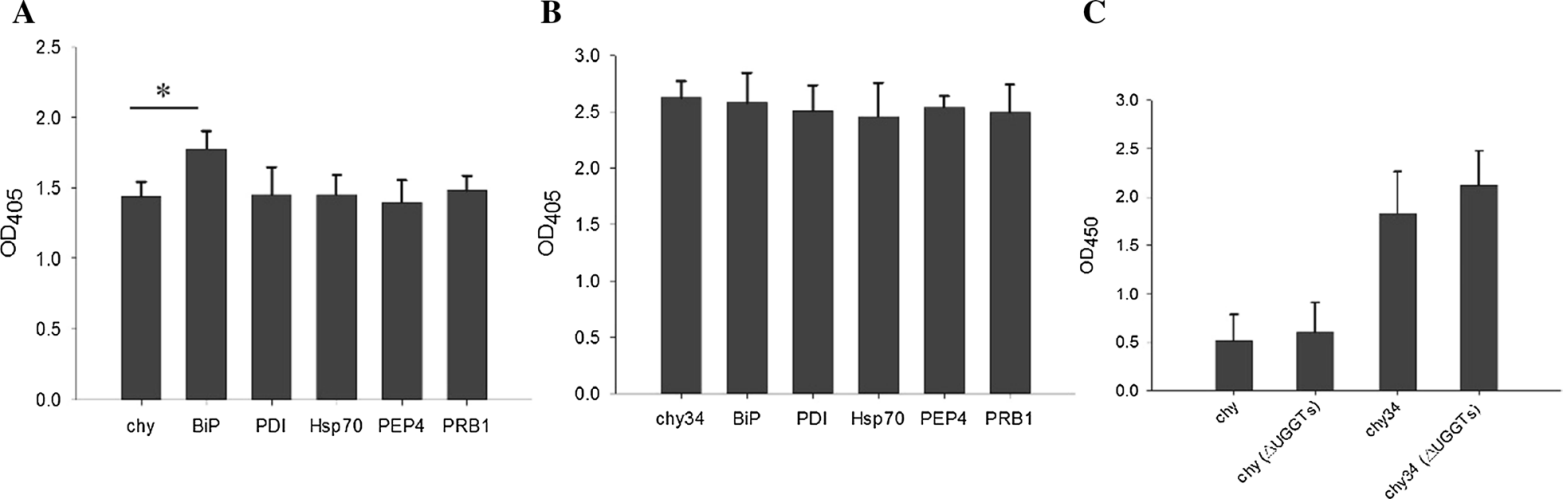
The effect of five DIPs co-expression and UGGTs knockout on the secretion of prochymosin in P. pastoris. **A** The effect of five DIPs co-expression on the secretion of chy; **B** The effect of five DIPs co-expression on the secretion of chy34; **C** The effect of UGGTs knockout on the secretion of chy and chy34. Data are presented as the mean ± SD from three independent experiments, *P < 0.05

In the ER related “protein processing in ER” pathway (Fig. 3), the proteins interacting with chy are significantly up-regulated, we speculate that these proteins may affect the secretion of chy. Therefore, five up-regulated proteins PDI and BiP in ER, PEP4 (Gene symbol: pas_chr3_1087), PRB1 (Gene symbol: pas_chr1-1_0226) in autophagy-yeast pathway (ppa04138) and ERAD related protein Hsp70 (Gene symbol: pas_chr3_0230) were selected for co-expression to study whether these proteins has an effect on the secretion of chy. The results showed that BiP increased the secretion of chy. Other four proteins had no effect. All six proteins had no significant effect on the secretion of chy34 (Fig. 4A, B).

### The knockout of UGGTs did not affect the secretion of chy and chy34

The above studies show that N-glycosylation changes the interacting proteins of prochymosin in ER. We speculated that there are elements that recognize N-glycosylation modification in ER. Previous studies have shown that, in other species, UGGTs (including two isoforms: UGGT1 and UGGT2) can recognize the folding state of glycoproteins [12, 13]。However, in this study, no UGGTs proteins were detected in the mass spectrometry results of the two samples.

This suggests that the UGGTs may not be the recognition element of prochymosin protein folding, or there may be other intermediate signal molecules. In order to study the role of UGGTs in prochymosin secretion pathway, we knocked out UGGT1 and UGGT2 in host cells (Additional file 1: Fig. S3). The knockout of UGGT1 and UGGT2 did not affect the growth of yeast cells (Additional file 1: Fig. S4). The genes of chy and chy34 were transformed into GS115 (△UGGTs) strain, and their secretion levels did not change significantly (Fig. 4C).

### Discussion

Nowadays, many eukaryotic expression systems have been commercialized [37], which including monoclonal antibodies production in mammalian cells [38], some viral vaccines production in baculovirus expression vector system [39], hepatitis B vaccine production in *Saccharomyces cerevisiae* [40] and insulin glargine production in *P. pastoris* [37], all these expression systems have the ability of N-glycosylation modification. More than half of all eukaryotic proteins are glycoproteins, and about 90% of them are N-glycosylated [10]. N-glycosylation exerts many biological functions in organisms. Among them, increasing glycoprotein secretion is one of the important functions. However, as the studies on the mechanisms of N-glycosylation affecting glycoproteins secretion, specially the bioinformatics analysis of intracellular relevant proteins were fewer, there are no reports on the application of N-glycosylation increasing protein expression to the industrial reality so far. Present study provided a preliminary research basis for the above question.

### N-glycosylation affects prochymosin protein properties

This study found that N-glycosylation increased the secretion of prochymosin, and the molecular weight and thermostability of the protein also increased significantly. Petrescu et al. reported that, oligosaccharide chains can reduce the conformational freedom of the Asn residue side chain and increase the structural rigidity of nearby amino acids, making them less susceptible to thermal denaturation [28]. Additionally, the key of the influence of oligosaccharide chain on the stability of protein structure lies in the N-glycosylated position, not the length of the oligosaccharide chain. At present, the main view is the oligosaccharide chains are hydrophilic macromolecules, and their presence makes it more difficult for glycoprotein to form a precipitate in an aqueous solution. Our research is consistent with these views, N-glycosylation significantly improved the thermostability of prochymosin. However, not all N-glycosylation of proteins can improve secretion, it may be that these N-glycosylation positions have no significant effect on thermostability, or only were weak glycosylated [29] or oligosaccharide chain was located inside the protein molecule. More experimental data are needed to explain these opposite phenomenon. On the other hand, Western-blot results (Fig. 1B) showed that after N-glycosylation at position 34, the molecular weight increased significantly, indicating that the protein was hyper-glycosylated. The hyper-glycosylation process occurred in the Golgi apparatus [30]. However, it has been found that the Golgi apparatus does not determine the secretion of glycoproteins [31]. Therefor, ER is the key organelle affecting prochymosin secretion.

### N-glycosylation changes intracellular proteins interacting with prochymosin

IP-MS results showed that the proteins interacting with chy were significantly more than that with chy34 (Fig. 2A, B), and all DIPs was up–regulated in chy sample. According to the results of GO, KEGG and COG (Fig. 2C, D, E and Additional file 3: Table S2), DIPs are involved in protein synthesis, metabolism, energy production/transformation and homeostasis maintenance, it indicated that the secretion of chy needs more molecular chaperones, consumes more energy to maintain cell homeostasis. Due to its low thermostability, chy binds to BIP and PDI in ER, preventing it from entering the secretory pathway. We measured the intracellular proteins of the two host cells by Western–blot, and did not detect prochymosin (the results were not shown), and this implying that chy did not stay in ER, but quickly entered the degradation pathway. This phenomenon was also verified by the significant up-regulation of the interacting proteins (Hsp70, PEP4 and PRB1) that related to the degradation pathway. When N-glycosylation sites were added to the prochymosin, the interacting protein was down regulated. In ER, prochymosin was separated from BIP and PDI etc., and then entered the secretory pathway. Among the five DIPs derived from ER, yeast-autophagy and ERAD pathway, BiP can increase the secretion of chy, indicating that BiP plays an important role in the mediation of prochymosin metabolic pathway. BiP had no effect on the secretion of chy34, which also verified that BiP selectively interacted with chy. In ER, BiP can bind to newly synthesized polypeptides [32], prevent their aggregation and precipitation, also prevent misfolded polypeptides from leaving the ER and guide them into the degradation pathway [15, 16]. It is further inferred that chy is not easy to form correct folding in ER, but after added an N-glycosylation, it promoted protein folding and increased secretion.

### UGGTs is not a recognition element of the quality control system for prochymosin secretion

In many species, UGGTs is a folding sensor of glycoprotein quality control system in the ER [33–35]. However, in *P. pastoris*, there has been no any reports about UGGTs function identification. In order to explore the effect of UGGTs on the secretion of prochymosin, we knocked out UGGT1 and UGGT2 of *P. pastoris* genome. The knockout of UGGTs did not affect the growth of yeast cells, indicating that UGGTs is not necessary for cell growth under normal conditions. And then we determined the effect of UGGTs knockout on the secretion of chy and chy34. Surprisingly, UGGTs had no effect on the secretion of both glycoproteins. It indicates that in *P. pastoris*, N-glycosylation can significantly change the secretion of prochymosin even without UGGTs. So we inferred that UGGTs is not the key recognition element of glycoproteins in *P. pastoris*. In *S. cerevisiae*, there is no UGGTs gene in the genome [36], and there may be other components in its glycoprotein quality control system to regulate this pathway. Although there are UGGTs gene in *P. pastoris* genome, our study did not identify its key function on glycoprotein secretion. In *P. pastoris*, whether BiP is the sensor for glycoprotein quality control needs to be further determined. In addition, it is worth noting that this study is limited to the prochymosin, and whether other glycoproteins meet the findings of this study remains to be verified.

### Conclusion

In conclusion, N-glycosylation significantly increased the secretion of prochymosin in *P. pastoris*, and DIPs before and after adding N-glycosylation site were identified by IP-MS. The up-regulated DIPs interacting with chy mainly involved in the misfolded protein pathway. At the same time, it was also found that BiP can improve the secretion of chy, and UGGTs do not play a key role in the secretion of prochymosin. This study is conducive to promote the industrial application of chymosin and provide a research basis for the interpretation of the molecular mechanism of N-glycosylation affecting glycoprotein secretion.

## Materials and methods

### Strains, vectors, and reagents

The yeast expression vector pPIC9K and the yeast strain *P. pastoris* GS115 were obtained from Life Technologies, Inc. (Grand Island, NY, USA). *E. coli* competent cells were purchased from TransGen Biotech (Beijing, China). The restriction enzyme and Peptide N-Glycosidase F (PNGase F) were purchased from New England Biolabs (Ipswich, MA, USA). Skimmed milk powder was purchased from Sigma-Aldrich (St. Louis, MO, USA). All other chemicals and reagents, unless otherwise stated, were acquired from Sinopharm Chemical Reagent Beijing Co., Ltd. (Beijing, China).

### Construction of mutant prochymosin expression vector

The expression vector pPIC9k-chy with the wild prochymosin genetic sequence [7] was used as a polymerase chain reaction (PCR) template for site-directed mutagenesis. To construct the prochymosin mutant in which one conserved N-glycosylated site, Asn-Val-Ser, was introduced into the propeptide, the codon for 34th Asn replaced the Ala (Fig. 1A). The primers used in site-directed mutagenesis were listed in Table 1. The PCR products were cloned into the *P. pastoris* vector pPIC9K using a Seamless Assembly Cloning Kit (CloneSmarter, USA) to yield the expression vectors carrying mutant prochymosin genes. Wild (chy) and mutant (chy34) recombinant plasmids were linearized by *Sal* I and then electrotransformed into GS115 by a Bio-Rad Micropulser Electroporater (Bio-Rad, Hercules, CA, USA). Electrotransformation was performed according to the manufacturer’s protocol (Life Technologies, USA).

**Table 1:** Primers for the site mutation, qPCR and verified of UGGTs gene knockout

Primer name ^a^	Sequence (5′ to 3′)
chy-F	gggtatctctcgagaaaagacaccatcaccatcaccactccggtatcaccagaatcc
chy34-F	cttgcaaagacaacaatacaacgtctcctccaagtactcctccttgggtaaggtcgc
chy34-R	gcgaccttacccaaggaggagtacttggaggagacgttgtattgttgtctttgcaag
chy-R	gtctaaggcgaattaattcgcggccgcttagatggccttggccaaaccgac
qPCR-F	ttgagatccatgccaaccgt
qPCR-R	acgtcacccaagatccacaa
act-F	agtgttcccatcggtcgtag
act-R	ggtgtggtgccagatctttt
UGGT1-F	tggcgggattcgtcaaagat
UGGT1-R	aatgtgagcagcaaaacgcc
UGGT2-F	gccaactggttcaaaacccc
UGGT2-R	tttgactgcgcaactacagc

^a^The primers chy-F, chy34-F, chy34-R, chy-R were used to amplified the gene of chy34. The primer pair qPCR-F and qPCR-R were used to qRT-PCR of prochymosin transcription in both host cells chy-GS115 and chy34-GS115, the act-F and act-R as the primer pair of internal reference gene actin. The primer pair UGGT1-F/UGGT1-R and UGGT1-F/UGGT1-R were used to verify the knockout of UGGT1 and UGGT2, respectively

### Secretion and identification of prochymosin in *P. pastoris*

The positive clones were inoculated in 10 mL of BMGY (1% yeast extract, 2% peptone, and 4% glycerol) medium containing gradient concentration of G418 (0.1, 0.2, 0.4, and 0.8 mg/mL) and cultured at 28 °C and 200 rpm for 48 h. Fourteen clones that could resist 0.4 mg/mL of G418 for each transformation event were selected and cultured at 28 °C and 200 rpm/min in 10 mL of BMGY medium until the OD_600_ reached 2.0. The cells were collected and resuspended in 10 mL of BMMY (1% yeast extract, 2% peptone, 0.5% methanol, and 10 mM potassium phosphate buffer at pH 6.0) medium and then cultured at 28 °C and 200 rpm for 72 h. Moreover, 50 μL of 100% methanol were added every 24 h. The cultures were centrifuged at 10,000 rpm for 5 min, and the supernatants containing secreted proteins were collected. Then, SDS-PAGE or Western-blot were carried out to verify the proteins. ELISA was used to analyze the relative concentrations of chy and chy34 proteins. The methods of SDS-PAGE, Western-blot and Enzyme linked immunosorbent assay (ELISA) were described in [26]. The primary antibody was rabbit anti-prochymosin polyclonal antibody (prepared by Abclonal Technology, Wuhan, China) and the second antibody was goat anti-rabbit IgG conjugated to Alkaline Phosphatase (ab97072, Abcam, Cambridge, MA, USA). During the ELISA reaction, Alkaline Phosphatase reacted with the substrate BCIP/NBT, and the resulting compound has a maximum absorbance at 405 nm. The concentration difference between chy and chy34 samples was directly reflected by the OD_405_. The pre-test results showed that there was no significant difference in the ELISA and Western-blot of 14 samples (the results were not shown). Three samples were randomly selected from 14 samples came from each transformation event to calculated the standard deviation.

### Real-time quantitative PCR

Two milliliters of BMGY culture (OD_600_ was about 2.0) were used to collect cells by centrifugation at 10,000 rpm for 5 min. The total RNA extraction was performed according to the manufacturer’s guidelines of the RNA Purification Kit (Yuanpinghao Bio, Beijing, China). The cDNA synthesis was performed using the cDNA Synthesis SuperMix Kit (TransGen Biotech, Beijing, China). The quantitative real-time PCR (qRT-PCR) was performed using a Green qRT-PCR SuperMix Kit (TransGen Biotech, Beijing, China) on a QuantStudio 3 instrument (Thermo Fisher Scientific, USA). The actin gene of *P. pastoris* GS115 was the reference gene. The qRT-PCR primer pair were listed in Table 1. The relative expression at the mRNA level were calculated by the relative 2^–ΔΔCT^ method [41]. The ΔCT value of chy was taken as the control and calculated the mRNA relative expression of chy34.

### Enzymatic activity and thermostability assay of prochymosin proteins

The full-length prochymosin has no enzymatic activity. Only after the autocatalytic cleavage of the propeptide was completed, the mature chymosin can exert its clotting activity. Acidic conditions were required for the autocatalytic cleavage. In order to determine whether the N-glycosylated mutation of the propeptide affected its autocatalytic cleavage, the enzymatic activity was determined after acid-neutralization of the supernatant of the recombinant protein cultures. The enzymatic activity was analyzed as described previously with minor modifications [26]. Substrate was prepared by mixing skim milk powder (26% w/v) in 10 mmol/L of potassium phosphate buffer (pH 5.5). The chy and chy34 expression strains were induced with 0.5% methanol for 72 h, and the supernatants of the cultures were collected by centrifuged at 10,000 rpm for 5 min. Autocatalytic cleavage was performed at pH 2.0 by the addition of 1 M HCl and incubated at 25 °C for 2 h. Then the activated enzyme solution was adjusted the pH to 5.5 with 1 M NaOH. The enzyme reactions were carried out in 2 ml centrifuge tubes with 10 µL of enzyme solution and 490 µL of substrate at 37 °C. The milk clots were visualized by tilting the tubes.

The thermostability of prochymosin were assessed as described previously with minor modifications [26]. The enzyme solution was first incubated for 8 h at a range of temperatures (from 25 °C to 65 °C), and then the enzymatic activity was measured by the method mentioned above. An average value of three relative activities was taken for each sample.

### Determination of the structural characterization of prochymosin protein molecular

Far-ultraviolet (UV) circular dichroism (CD) spectroscopy was used to analyze the secondary structure fractions of chy and chy34. The supernatant of the culture was filtered by sterile filter unit with 0.45 μm PVDF membrane (Merck Millipore). Protein was purified by size exclusion chromatography with sephadex G75 column. SDS-PAGE and Western-blot determined the target proteins. The purified protein was dialyzed in 0.2 mg/mL in 20 mM sodium phosphate buffer (pH 7.4) and concentrated by ultrafiltration membrane (Amicon Ultra-15 Centrifugal Filter Unit, Merck-Millipore). Far-UV CD spectra were taken on a Chirascan Plus (Applied Photophysics) with the cell of 0.5 mm pathlength. Measurement was performed at 25 °C in the range from 190 to 260 nm. The sodium phosphate buffer was used as blank control. An average of three times scans was taken for each sample. Pro-Data Viewer and CDNN software was used to analyze the CD spectra result and calculate secondary structure fractions.

Hitachi F-7000 fluorescence spectrophotometer (Hitachi) was used to analyze the change of tertiary structure of proteins. Hydrophobic tryptophan was the intrinsic fluorescent group of protein. The tryptophan much more tends to be buried in the inside hydrophobic area of protein molecule, the more the maximum emission peak of the protein tends to blue shift and the lower the fluorescence density, which indicates that the protein molecule has more thermostability [23]. The purified protein sample of 0.1 mg/mL was taken for the fluorescence spectra and the data were collected by excitation at 280 nm and had an emission range of 280–400 nm. Slit widths were set at 5 nm with a scan speed of 12,000 nm/minute.

### Immunoprecipitation and label-free quantification mass spectrometry analysis

After 24 h of methanol induction, the cells were collected by centrifuge at 10,000 rpm for 5 min. The cell lysate and immunoprecipitation were performed according to the manufacturer’s protocol of Immunoprecipitation Kit (Sangon Biotech, Shanghai, China). The antibody used for immunoprecipitation was rabbit anti-prochymosin polyclonal antibody (prepared by Abclonal Technology, Wuhan, China). After immunoprecipitation, the proteins were digested by trypsin. Samples from three independent biological repetitions were mixed and used for mass spectrometry analysis. Equal amounts of digested chy and chy34 sample were separated by a liquid chromatography UltiMate 3000 RSLCnano (Thermo Scientific) with capture column (3 μm, 120 Å, 100 μm × 20 mm) and analytical column (2 μm, 120 Å, 75 μm × 150 mm). The flow rate of the liquid phase is set to 300 nL/min using a gradient buffer formed by Solvent A (3% DMSO, 0.1% formic acid, 97% H_2_O) and Solvent B (3% DMSO, 0.1% formic acid, 97% ACN). Separated peptide fragments were indentified using a Q Exactive Plus spectrometer (Thermo Scientific). Each scan cycle consists of one MS full scan (R = 70 K, AGC = 3e6, max IT = 20 ms, scan range = 350–1800 1 m/z) and followed by 15 MS / MS scans (R = 17.5 K, AGC = 2e5, max IT = 100 ms). The MS/MS spectra was searched against the UniProt database (https://www.uniprot.org) by use of ProteinPilot (V4.5) with Unused ≥ 1.3 for protein identification.

### Bioinformatics

The difference interacted proteins (DIPs) were screened based on the number of spectra of each protein. Calculate the ratio and the average value of the protein spectra (MeanSP) in different samples, x = log2 ratio, y = log2 MeanSP, The DIPs screening boundary line y = C / (x- × 0). Proteins distributed outside the boundary line were labeled as DIPs. DIPs with spectra difference more than 1.5-fold were labeled “ ± ” (the boundary lines were y1 and y3), the spectra difference more than twofold were labeled “ +  ± -” (the boundary lines were y2 and y4). In this study, all proteins distributed outside the boundary line y1 and y3 were selected for subsequent analysis (Additional file 1: Fig S1C). Gene Ontology (GO), Kyoto Encyclopedia of Genes and Genomes (KEGG) and Cluster of Orthologous Groups of proteins (COG) annotation based on ftp://ftp.ncbi.nih.gov/gene/DATA/gene2go.gz, http://www.genome.jp/kegg and http://eggnogdb.embl.de/, respectively. The GO and KEGG pathway enrichment analyses were performed using the R software.

### Interacting proteins co-expressed with prochymosin in *P. pastoris*

The DIPs PDI (Gene symbol: PAS_chr1-1_0160), BiP (Gene symbol: PAS_chr2-1_0140), Hsp70 (Gene symbol: PAS_chr3_0230), PEP4 (Gene symbol: PAS_chr3_1087) and PRB1 (Gene symbol: PAS_chr1-1_0226) were selected to co-expressed with prochymosin in *P. pastoris*. Fig. S2 depicts the plasmid vector structure carrying various interacting protein gene (co-expression vector). The restriction enzyme *Eco*R I is the location of insertion of interacting proteins. Vectors carrying different interacting protein genes were electrotransformed into strains chy-GS115 and chy34-GS115, respectively. Three positive clones were inoculated for each transform event and were cultured in 10 mL of BMGY with 0.1 mg/mL of zeocin and cultured at 28 °C and 200 rpm for 72 h. The cells were collected and resuspended in BMMY with 0.1 mg/mL of zeocin. The cultures were induced by 0.5% (v/v) methanol for 72 h. The supernatants were collected and used for comparing secretion levels of prochymosin by ELISA. The prime antibody and the second antibody were rabbit anti-prochymosin polyclonal antibody and goat anti-rabbit IgG conjugated to AP, respectively.

### Knockout of UGGT1 and UGGT2 genes by CRISPR/Cas9

Knockout of UGGT1 (GenBank: NC_012965.1) and UGGT2 (GenBank: NC_012963.1) genes of *P. pastoris* GS115 by CRISPR/Cas9 was according to [27]. The sgRNA was designed for the target site in the website E-CRISP (http://www.e-crisp.org/E-CRISP/designcrispr.html). The target sites were located at 800–819 bp of UGGT1 gene and 344–363 bp of the UGGT2 gene. Gene knockout was verified by sequencing PCR products with genome as template, the sequencing primers were listed in Table 1.

## Supplementary Information

**Additional file 1: Fig. S1** Polypeptide quality monitoring and differential interacting proteins screening data. **A** Peptide length distribution after trypsin digestion; **B** The number of spectra, peptides and proteins interacting with chy and chy34; **C** Distribution of differential interacting proteins (DIPs), red means the spectra difference more than 2-fold, yellow means the spectra difference more than 1.5-fold, grey means no difference. **Fig. S2** Map of the co-expression vector used to transformation of five DIPs into chy-GS115 or chy34-GS115 cell. pPFK indicates the promoter of 6-phosphofructo-1-kinase gamma-subunit from P. pastoris, AOX1 terminator indicates the alcohol oxidase transcriptional termination region, EcoRIis the integration site of DIPs. BleoR indicates the zeocin resistant gene, CEN6_ARS indicates the yeast origin of replication, ori indicates the pUC origin. **Fig. S3** Verification of UGGT1 and UGGT2 knockout. A/C Sequence alignment, Sbjct and Query indicate the UGGT1/UGGT2 gene sequence before and after knockout. B/D Sequencing peak map of UGGT1/UGGT2 gene after knockout. The triangle indicate the deletion site. **Fig. S4** Growth curve acquisition of GS115, chy-GS115 and chy34-GS115 strains, ∆UGGTs indicates that UGGT1 and UGGT2 have been knocked out. **Fig. S5** **A** Far-UV CD spectra of wild and mutant prochymosins. The red line means the CD spectra of chy and the blue one was of the chy34. The table below showed the percentage of the protein’s secondary structure of chy and chy34. **B** Fluorescence spectra of chy and chy34

**Additional file 2:** **Table S1.** Protein identification and abundance of differential interacting proteins with chy and chy34

**Additional file 3.** Classification of DIPs according to GO, KEGG and COG enrichment methods.

## Data Availability

All data generated or analysed during this study are included in this published article.
