# Supplementary material for: The introduction of an N-glycosylation site into prochymosin greatly enhances its production and secretion by Pichia pastoris
Source: Microb Cell Fact. 2022 Aug 30;21:177. doi: 10.1186/s12934-022-01904-3 (PMC9429577; doi:10.1186/s12934-022-01904-3)
Supplement: Supplementary file 1 — Additional file 1: Fig. S1 Polypeptide quality monitoring and differential interacting proteins screening data. A Peptide length distribution after trypsin digestion; B The number of spectra, peptides and proteins interacting with chy and chy34; C Distribution of differential interacting proteins (DIPs), red means the spectra difference more than 2-fold, yellow means the spectra difference more than 1.5-fold, grey means no difference. Fig. S2 Map of the co-expression vector used to transformation of five DIPs into chy-GS115 or chy34-GS115 cell. pPFK indicates the promoter of 6-phosphofructo-1-kinase gamma-subunit from P. pastoris, AOX1 terminator indicates the alcohol oxidase transcriptional termination region, EcoRIis the integration site of DIPs. BleoR indicates the zeocin resistant gene, CEN6_ARS indicates the yeast origin of replication, ori indicates the pUC origin. Fig. S3 Verification of UGGT1 and UGGT2 knockout. A/C Sequence alignment, Sbjct and Query indicate the UGGT1/UGGT2 gene sequence before and after knockout. B/D Sequencing peak map of UGGT1/UGGT2 gene after knockout. The triangle indicate the deletion site. Fig. S4 Growth curve acquisition of GS115, chy-GS115 and chy34-GS115 strains, ∆UGGTs indicates that UGGT1 and UGGT2 have been knocked out. Fig. S5 A Far-UV CD spectra of wild and mutant prochymosins. The red line means the CD spectra of chy and the blue one was of the chy34. The table below showed the percentage of the protein’s secondary structure of chy and chy34. B Fluorescence spectra of chy and chy34 [file 12934_2022_1904_MOESM1_ESM.docx]

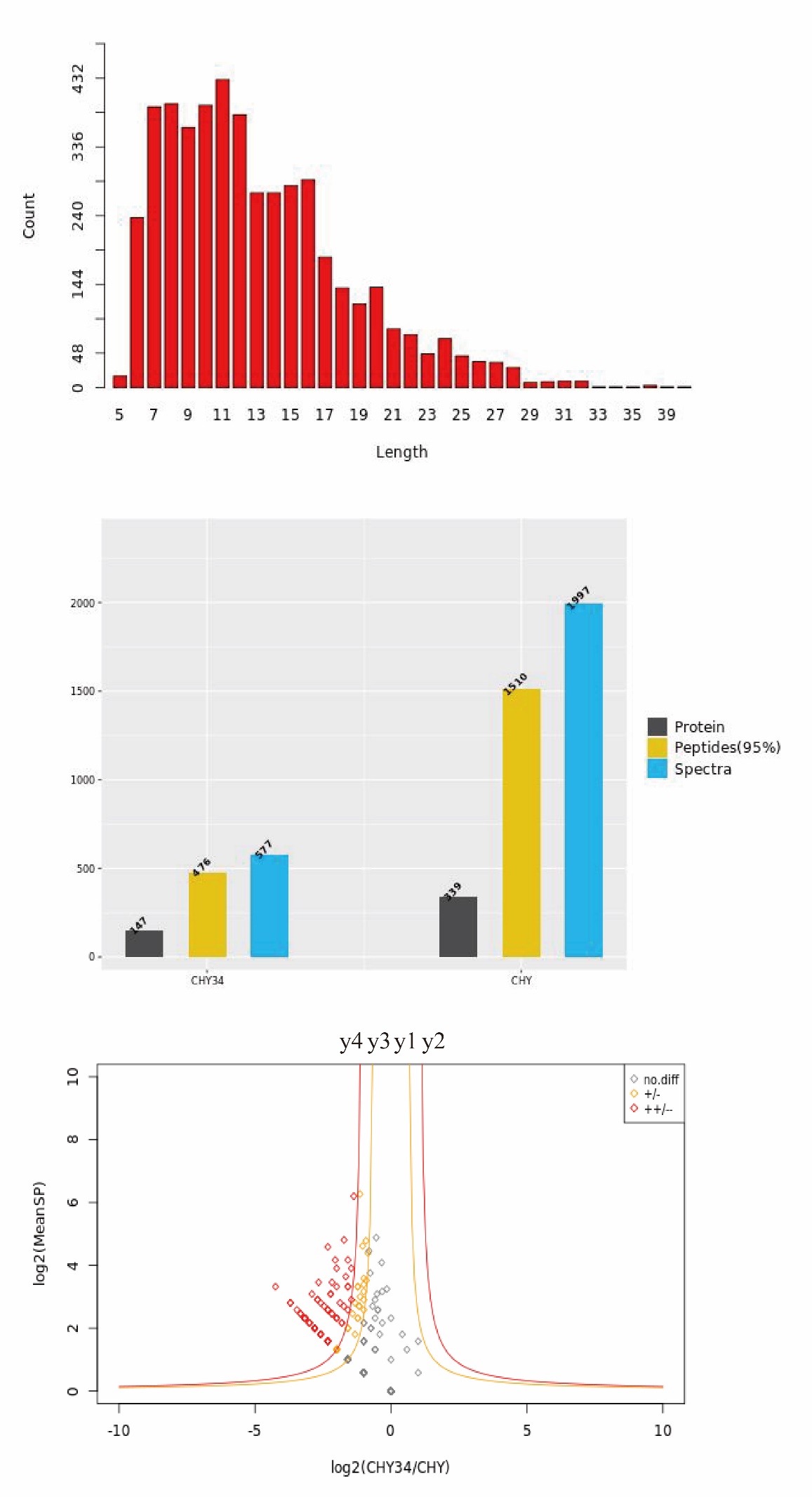


A

B

C

Fig. S1 Polypeptide quality monitoring and differential interacting proteins screening data. **A** Peptide length distribution after trypsin digestion; **B** The number of spectra, peptides and proteins interacting with chy and chy34; **C** Distribution of differential interacting proteins (DIPs), red means the spectra difference more than 2-fold, yellow means the spectra difference more than 1.5-fold, grey means no difference.


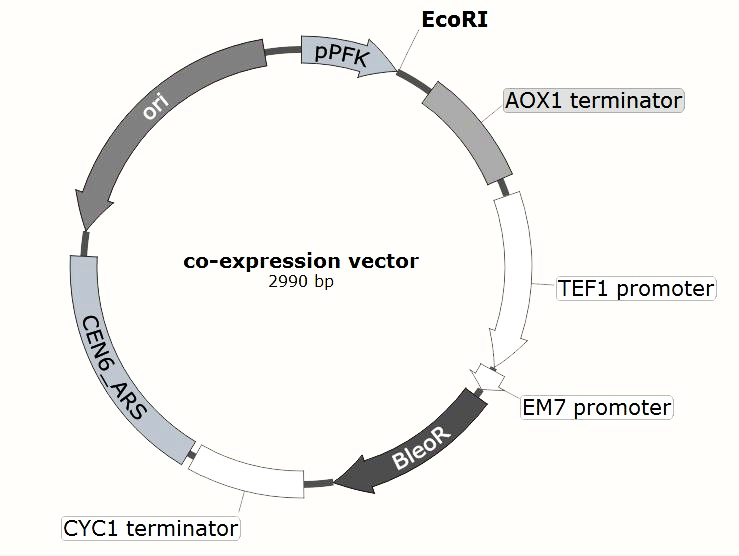


Fig. S2 Map of the co-expression vector used to transformation of five DIPs into chy-GS115 or chy34-GS115 cell. pPFK indicates the promoter of 6-phosphofructo-1-kinase gamma-subunit from *P. pastoris*, AOX1 terminator indicates the alcohol oxidase transcriptional termination region, *EcoR*Ⅰis the integration site of DIPs. BleoR indicates the zeocin resistant gene, CEN6_ARS indicates the yeast origin of replication, ori indicates the pUC origin.


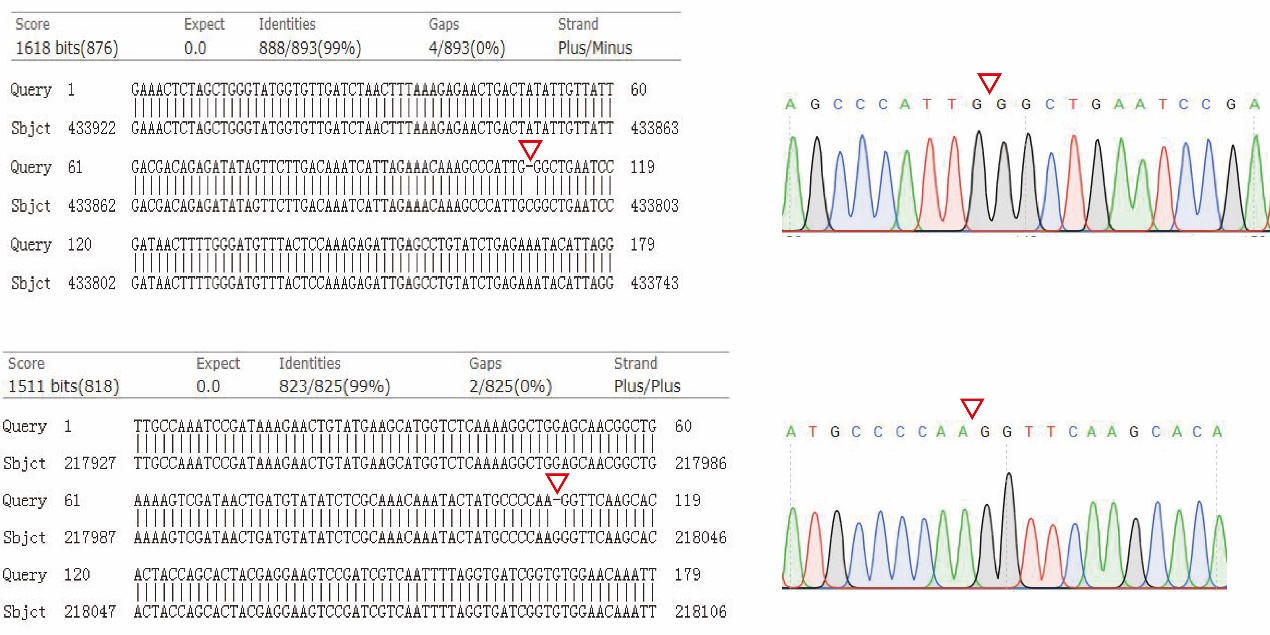


A

B

C

D

Fig. S3 Verification of UGGT1 and UGGT2 knockout. A/C Sequence alignment, Sbjct and Query indicate the UGGT1/UGGT2 gene sequence before and after knockout. B/D Sequencing peak map of UGGT1/UGGT2 gene after knockout. The triangle indicate the deletion site.


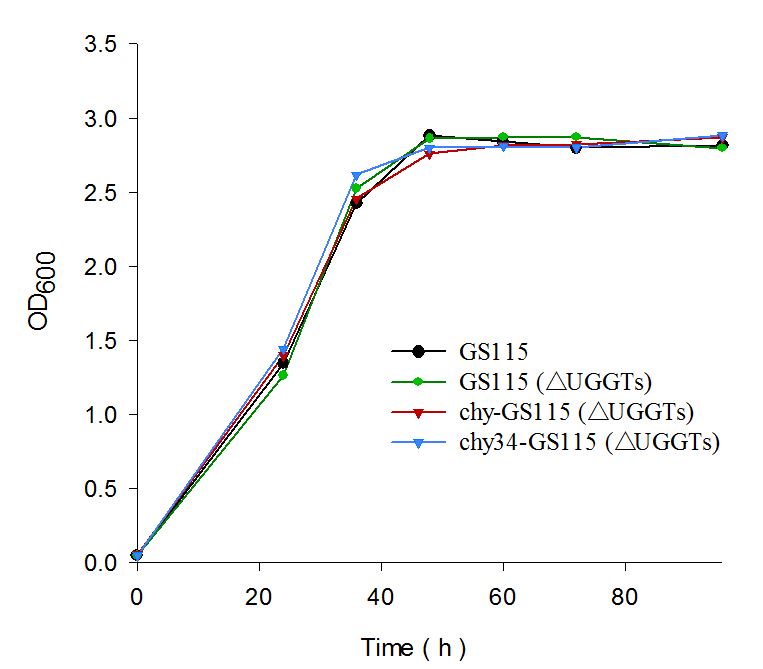


Fig. S4 Growth curve acquisition of GS115, chy-GS115 and chy34-GS115 strains, △UGGTs indicates that UGGT1 and UGGT2 have been knocked out.


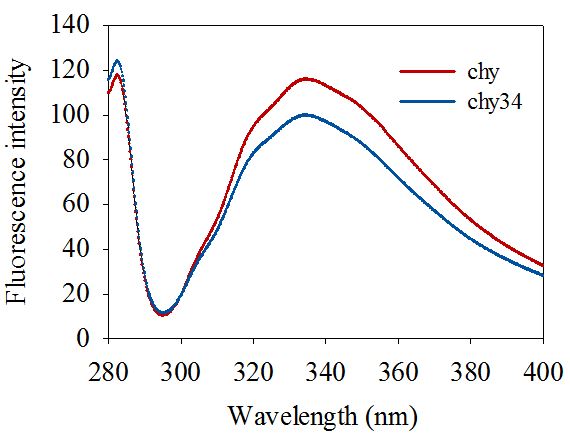

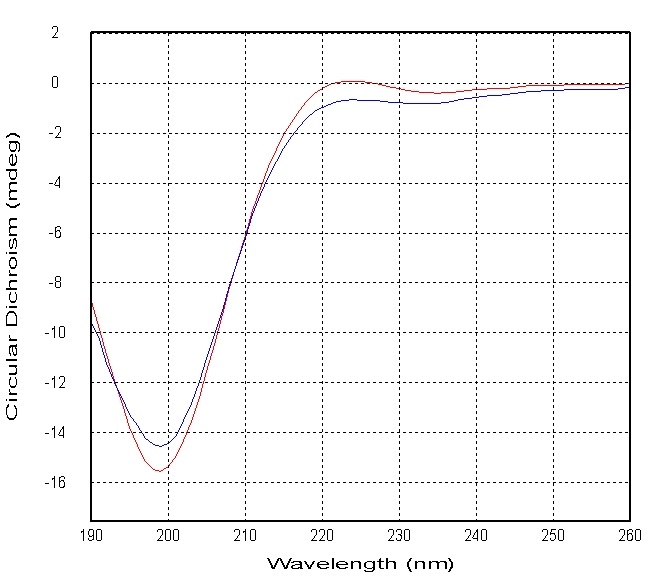


A

B

| prochymosin | Helix (%) | Strand (%) | Turn (%) | Randomcoil (%) |
| --- | --- | --- | --- | --- |
| chy | 4.3 | 12.9 | 31.6 | 50.8 |
| chy34 | 4.5 | 12.6 | 31.1 | 51.3 |

Fig. S5 **A** Far-UV CD spectra of wild and mutant prochymosins. The red line means the CD spectra of chy and the blue one was of the chy34. The table below showed the percentage of the protein’s secondary structure of chy and chy34. **B** Fluorescence spectra of chy and chy34.
